# Model for End-Stage Liver Disease Including Na, Age, and Sex Is Powerful Predictor of Survival in COVID-19 Patients on Extracorporeal Membrane Oxygenation

**DOI:** 10.3390/diagnostics14171954

**Published:** 2024-09-04

**Authors:** Freya Sophie Jenkins, Mohammed Morjan, Jan-Philipp Minol, Esma Yilmaz, Ismail Dalyanoglu, Moritz Benjamin Immohr, Bernhard Korbmacher, Udo Boeken, Artur Lichtenberg, Hannan Dalyanoglu

**Affiliations:** 1 Department of Cardiac Surgery, University of Dusseldorf, 40225 Dusseldorf, Germany; freyasophie.jenkins@med.uni-duesseldorf.de (F.S.J.); mohammed.morjan@med.uni-duesseldorf.de (M.M.); esma.yilmaz@med.uni-duesseldorf.de (E.Y.); moritz.immohr@uk-essen.de (M.B.I.); korbmacher@uni-duesseldorf.de (B.K.); boeken@uni-duesseldorf.de (U.B.); artur.lichtenberg@med.uni-duesseldorf.de (A.L.); hannan.dalyanoglu@med.uni-duesseldorf.de (H.D.); 2 Medical Faculty, Semmelweis University, 1085 Budapest, Hungary

**Keywords:** COVID-19, ECMO, predictors, MELD, in-hospital mortality

## Abstract

Aim: Extracorporeal membrane oxygenation (ECMO) is resource-intensive, is associated with significant morbidity and mortality, and requires careful patient selection. This study examined whether the model for end-stage liver disease (MELD) score is a suitable predictor of in-hospital mortality in patients with COVID-19. Materials and Methods: We retrospectively assessed patients with COVID-19 on ECMO at our institution from March 2020 to May 2021. MELD scoring was performed using laboratory values recorded prior to ECMO initiation. A multiple logistic regression model was established. Results: A total of 66 patients with COVID-19 on ECMO were included (median age of 58.5 years; 83.3% male). The in-hospital mortality was 74.2%. In relation to mortality, patients with MELD Na scores >13.8 showed 6.5-fold higher odds, patients aged >53.5 years showed 18.4-fold higher odds, and male patients showed 15.9-fold higher odds. The predictive power of a model combining the MELD Na with age and sex was significant (AUC = 0.883, *p* < 0.001). The findings in the COVID-19 patients were not generalizable to a group of non-COVID-19 patients on ECMO. Conclusions: A model combining the MELD Na, age, and sex has high predictive power for in-hospital mortality in patients with COVID-19 on ECMO, and it may be clinically useful for guiding patient selection in critically ill COVID-19 patients both now and in the future, should the virus widely re-emerge.

## 1. Introduction

Extracorporeal membrane oxygenation (ECMO) substitutes for pulmonary function through oxygenation and decarboxylation of blood in an extracorporeal circuit. It is used in the management of acute respiratory distress syndrome (ARDS) when conventional ventilation and therapy, including optimal fluid balance and prone positioning where appropriate, are insufficient to maintain adequate gas exchange, with subsequent life-threatening hypoxemia or hypercapnia [1].

An indication for ECMO is generally independent of the underlying etiology, and it has been widely used to manage patients with respiratory failure associated with coronavirus disease 2019 (COVID-19) [2,3]. While ECMO can be lifesaving for some COVID-19 patients, it is also associated with morbidity and mortality, both in the short and long term. Previous studies have shown that COVID-19 patients who require ECMO tend to experience more severe physical and psychological long-term effects of the disease compared to those who do not need ECMO support [4,5]. Robust predictors of outcomes are important for guiding patient selection, as in addition to patient morbidity and mortality, ECMO is also resource-intensive.

Several scoring systems have been developed to predict the survival of patients receiving ECMO for ARDS such as the ECMOnet score [6], the PRESERVE (PRedicting dEath for SEvere ARDS on VV-ECMO) score [7], the RESP (Respiratory ECMO Survival Prediction) score [8], the Roch score [9], and the PRESET (PREdiction of Survival on ECMO Therapy) score [10]. These scores each combine a different variety of clinical, ventilation-associated, and laboratory-based data and have been shown to have good discriminatory power in their respective cohorts. However, these scores also have limitations. For example, some do not reproducibly discriminate based on mortality and are not generalizable across underlying ARDS pathologies; e.g., the RESP score has been shown to predict survival in non-COVID-19 patients with ARDS but not in patients with COVID-19 [11]. Prognostic scoring systems are also likely to achieve improved discriminatory power when considering measures of multiorgan failure, which is commonly seen in patients with COVID-19 and ARDS.

Therefore, there is a need for further scoring systems based on easily available parameters in COVID-19 patients with ARDS that are on ECMO, including measures of renal and hepatic dysfunction.

The model for end-stage liver disease (MELD) score was originally developed to predict survival in liver transplant patients, and it incorporates renal and hepatic dysfunction [12]. The MELD score has undergone several modifications from the original version covering bilirubin, INR, and creatinine to a version including sodium (“MELD Na”) [13] and a version excluding INR for patients on anticoagulant therapy (“MELD XI”) [14] (see Table 1). The MELD scoring system has shown associations with outcomes in patients undergoing cardiac surgery, critically ill patients in intensive care units, and patients on venoarterial and venovenous ECMO [15,16,17,18,19,20,21]. The prognostic value of the MELD in COVID-19 patients on ECMO has not previously been reported.

The aim of the current study was to examine whether the MELD score is a good predictor of in-hospital mortality in patients with COVID-19 undergoing ECMO for ARDS.

## 2. Materials and Methods

### 2.1. Study Patients

This study included patients with COVID-19 that required ECMO at our institution between March 2020 and May 2021. A separate group of non-COVID-19 patients with ARDS on ECMO was identified to assess the broader applicability of the findings in the COVID-19 patients. The non-COVID-19 patients were not matched to the COVID-19 patients to ensure a typical representation of ARDS with a cause other than COVID-19 requiring ECMO at our institution.

### 2.2. Study Design

This single-center study was retrospective in its design. All data were available and retrievable prior to the start of the study and were anonymized and organized in numerical order for analysis. MELD scoring was performed using laboratory values recorded prior to ECMO initiation.

### 2.3. Ethics

This study followed the principles of the Declaration of Helsinki and was approved by the Ethics Committee of Heinrich Heine University, Dusseldorf (study number: 2021—1630_1).

### 2.4. Data Analysis

Descriptive statistics for normally distributed quantitative variables are summarized as means and standard deviations. For skewed data, medians and interquartile ranges are shown. Categorical data are presented as proportions. Spearman’s rank correlation was used to analyze the relationships among the quantitative variables. The effects of the variables on in-hospital mortality were evaluated using a stepwise approach. In the initial analysis, the chi-squared test, the Mann–Whitney U test, and Student’s *t* test were used to assess the raw differences between the survivors and non-survivors. For statistically significant quantitative variables, receiver operating characteristic (ROC) curves were used for discriminatory power, with the corresponding area under the curve (AUC) analyzed with the Mann–Whitney U test and the optimal cut-off values identified using Youden’s index.

Statistically significant variables were combined into a multivariate logistic regression model for COVID-19 patients for further analysis. The model’s accuracy was assessed by comparing the predicted outcomes to the actual outcomes. The model was applied to a control group to evaluate its applicability to non-COVID-19 patients on ECMO. Statistical significance was established as *p* < 0.05. Analysis and evaluation of the data were carried out using the statistical software R, version 4.3.2 (R Foundation for Statistical Computing, Vienna, Austria).

## 3. Results

### 3.1. COVID-19 Patients

#### 3.1.1. Patient Characteristics

A total of 66 patients with COVID-19 on ECMO were included in the analysis (median age of 58.5 years; IQR of 51–61 years; 83.3% male). Most of the patients were overweight, with a median BMI of 29.0 (IQR of 26–32). One in ten patients had an official diagnosis of chronic obstructive pulmonary disease (COPD). Smoking was uncommon, with only 4.5% of the patients being active smokers (see Table 2).

#### 3.1.2. MELD Scores

The median scores on admission were 12.6 for the MELD, 13.7 for the MELD Na, and 12.1 for the MELD XI. Almost one-third of the patients (30.3%) had a MELD score greater than 20. The different MELD scores showed good concordance with each other, with the MELD and MELD Na showing the best correlation. None of the patient characteristics were associated with the MELD score, including sex (*p* = 0.085), age (Spearman’s ρ = −0.024, *p* = 0.200), BMI (Spearman’s ρ = −0.190, *p* = 0.120), and the time between symptom onset and ECMO implementation (Spearman’s ρ = −0.026, *p* = 0.840). No significant associations were observed between these variables and the MELD Na and MELD XI scores.

#### 3.1.3. ECMO and Weaning

The median time between the onset of symptoms and ECMO implementation was 18.0 days (IQR of 11–21 days) (see Table 2). The drainage cannulas for ECMO were in the right- or left-sided femoral veins in all 66 patients. The return cannulas were in the right jugular vein in 46 (69.7%) patients, in the right or left femoral veins in 19 (28.8%) patients, and in the right subclavian vein in 1 (1.5%) patient. Of the 66 patients, 12 (18.2%) required an arterial return cannula in the right or left femoral arteries for a veno-arteriovenous ECMO configuration to address hemodynamic instability in addition to continuing respiratory failure. Weaning from ECMO was achieved in 23 (34.8%) patients after a median ECMO duration of 13.0 days (IQR of 8–35 days). The overall median time on ECMO for all patients was 11.0 days (IQR of 5–27 days).

#### 3.1.4. In-Hospital Mortality

Of the 66 patients, 49 (74.2%) died in hospital, with the remaining 17 patients being discharged. Among the 49 non-survivors, 6 had been weaned off ECMO and survived a median of 4.5 days post-ECMO (IQR of 3–11 days). The main causes of death included respiratory failure, sepsis, multiorgan failure, and massive cerebral hemorrhage. In the univariate analysis, compared to the survivors, the patients who died in hospital were more often male, were older (59.0 vs. 50.0 years, *p* = 0.030), had higher pre-ECMO MELD scores, and had higher serum creatinine levels on admission (see Table 3). There were no differences in recorded comorbidities between the survivors and non-survivors, including diabetes, hyperlipidemia, hypertension, chronic obstructive pulmonary disease, and smoking history. The time between symptom onset and ECMO implementation was not associated with mortality (18.0 vs. 17.0 days, *p* = 0.950). Neither was the time on invasive mechanical ventilation before initiation of ECMO therapy (3.0 vs. 3.5 days, *p* = 0.842). The duration of ECMO therapy was not different between the survivors and non-survivors (*p* = 0.106). The addition of an arterial cannula, resulting in a veno-arteriovenous ECMO configuration, was not associated with a higher risk of in-hospital mortality compared to a solely venovenous ECMO configuration (*p* = 0.158).

**Figure 1:**
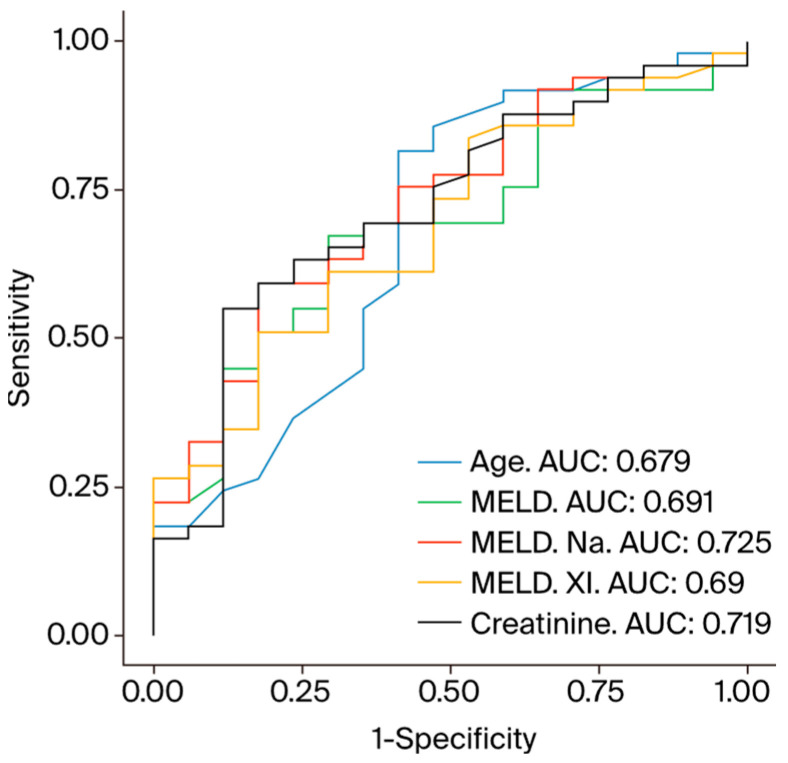
ROC curves for in-hospital mortality. Legend: AUC = area under the curve. MELD = model for end-stage liver disease. Na = sodium. ROC = receiver operating characteristic.

The MELD, MELD Na, MELD XI, age, and serum creatinine values showed good discriminatory power for in-hospital mortality in the ROC curve analysis (MELD AUC = 0.691, *p* = 0.010; MELD Na AUC = 0.725, *p* = 0.003; MELD XI AUC = 0.690, *p* = 0.010; age AUC = 0.679, *p* = 0.014; serum creatinine AUC = 0.719, *p* = 0.004) (see Figure 1). The optimal cut-off values for the quantitative predictor variables are shown in Table 4.

**Figure 2:**
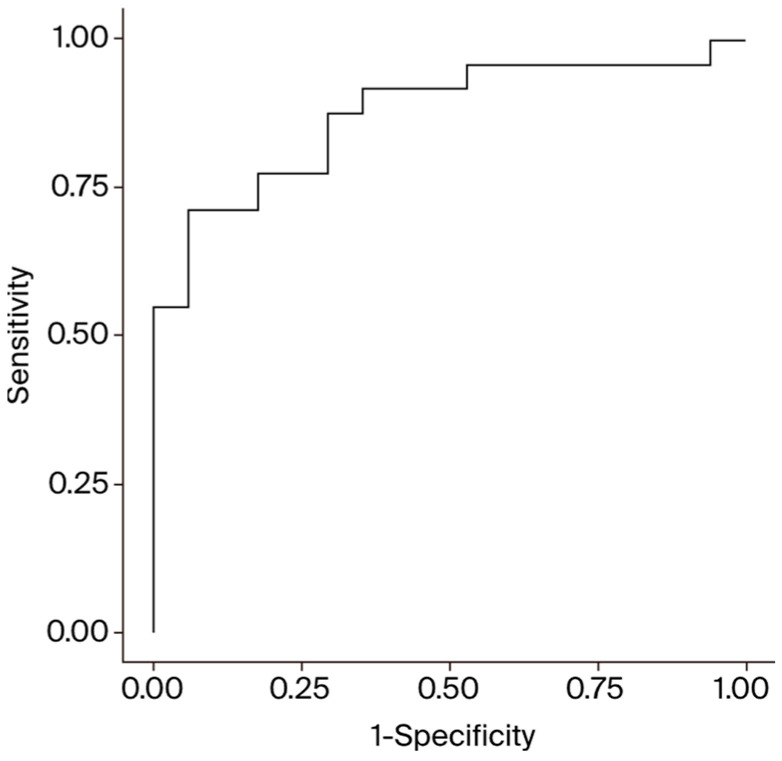
ROC curve for the combined model including the MELD Na, age, and gender for prediction of in-hospital mortality. Area under the curve: 0.883.

In the multivariate analysis, the MELD Na, age, and sex remained significant, while the other variables did not. Patients with MELD Na scores above the cut-off value of 13.8 had 6.5-fold higher odds of dying in hospital (95% CI = 1.2–36.3), patients older than 53.5 years had 18.4-fold higher odds of dying in hospital (95% CI = 3.1–108.3), and male patients had 15.9-fold higher odds of dying in hospital (95% CI = 2.4–104.8). The accuracy of the logistic regression model was 0.758 for age alone, 0.788 for sex alone, and 0.742 for the MELD Na alone. Combining all three variables into one multiple logistic regression model achieved an AUC for in-hospital mortality of 0.883 (*p* < 0.001) (see Figure 2). This was higher than with any variable alone, any combination of two variables, and models using the MELD or MELD XI in lieu of the MELD Na, with those scores both achieving accuracy values of 0.818.

### 3.2. Non-COVID-19 Patients

A total of 32 patients on ECMO for ARDS with a cause other than COVID-19 were used to assess the broader applicability of the findings from the COVID-19 patients.

The non-COVID-19 patients had lower BMI values (26.3 vs. 29.0, *p* = 0.010), were younger (51.5 vs. 58.5 years, *p* = 0.010), more frequently had histories with prior myocardial infarctions (18.8% vs. 1.5%, *p* = 0.010) and smoking (34.4% vs. 4.5%, *p* < 0.01), and spent less time being symptomatic before receiving ECMO (0.5 days vs. 18.0, *p* < 0.01). The non-COVID-19 patients had lower MELD scores, primarily due to lower serum creatinine levels upon admission (0.9 vs. 1.3, *p* = 0.002). There were no differences in the remaining MELD score components of bilirubin and the international normalized ratio (INR). The in-hospital mortality was comparable for the non-COVID-19 and COVID-19 patients (81.3% vs. 74.2%, *p* = 0.608).

The MELD XI showed significant discriminatory power for in-hospital mortality among the non-COVID-19 patients (AUC = 0.769, *p* = 0.022, cut-off value of 7.2) in the ROC curve analysis. In contrast to the COVID-19 patients, none of the measured variables achieved significance in the multivariate analysis of the ECMO patients without COVID-19, including age, sex, and MELD scores. Applying the COVID-19 model including the MELD Na, age, and sex to predict in-hospital mortality in this patient cohort achieved an accuracy of 0.562.

## 4. Discussion

This is the first report on the applicability of the MELD scoring system as a predictive factor for outcomes in COVID-19-related ARDS patients on ECMO. Our study’s main findings are as follows: First, the MELD scores prior to ECMO initiation, age, and sex independently predicted in-hospital mortality in COVID-19 patients on ECMO. Second, a model combining the pre-ECMO MELD Na with age and sex could be used to predict in-hospital mortality in patients with COVID-19-related ARDS on ECMO. To date, the published literature analyzing the use of the MELD scoring system for prognosis in ECMO patients is primarily focused on patients on venoarterial ECMO with cardiac failure, given this population’s propensity for multiorgan failure. Some studies on venoarterial ECMO patients have found good predictive values for the different MELD scores—MELD, MELD Na, and MELD XI—for short-term mortality and adverse events [22,23].

Two studies have specifically assessed MELD scoring in patients on venovenous ECMO. In their recent study, Sandrio and colleagues found MELD scoring to be a better predictor of mortality than other arguably more comprehensive scoring systems, including SOFA (Sequential Organ Failure Assessment), SAPS II (Simplified Acute Physiology Score), PRESERVE (PRedicting dEath for SEvere ARDS on VV-ECMO), and RESP (Respiratory Extracorporeal Membrane Oxygenation Survival Prediction) [21]. The authors reported a cut-off value of 16 for the original MELD score. In their study of 71 patients on venovenous ECMO, Watanabe and colleagues found the original MELD score to be predictive of in-hospital mortality and suggested its use in these patients alongside the RESP score, with a cut-off value of 12 for the original MELD score [17]. Watanabe’s study did not include COVID-19 patients, as it covered the period from 2016 to 2019. For Sandrio’s study, the period was January 2017 to December 2020, and it was not reported how many of the 187 ECMO patients had COVID-19.

Although COVID-19 primarily affects the respiratory system, it is considered a systemic disease with multiorgan involvement. Our study showed that multiorgan dysfunction, as captured quantitatively using the MELD scoring system, is predictive of in-hospital mortality in these patients, with cut-off values of 10.7 for the MELD, 13.8 for the MELD Na, and 13.9 for the MELD XI. In the context of COVID-19, the accuracy of MELD-based scores in predicting outcomes may differ from ARDS-specific scores such as RESP due to differences in the underlying pathophysiologies these scores account for. The MELD XI was associated with in-hospital mortality in ECMO patients without COVID-19 in a univariate analysis in our study. Combining the MELD Na, age, and sex into one multiple logistic regression model achieved the highest predictive power for in-hospital mortality in COVID-19 patients on ECMO, higher than for any variable alone, any combination of two variables, and models using the MELD or MELD XI in lieu of the MELD Na. Compared to the previously published scores for patients with respiratory failure on venovenous ECMO, for example the ECMOnet, PRESERVE, and RESP scores, our score achieved higher discriminatory power for mortality with an AUC of 0.883. Additional research is warranted in this area to explore associations between the MELD scoring system and other patient outcomes, such as post-COVID-19 conditions.

Interestingly, our study did not find an association between the timing of ECMO initiation relative to symptom onset and outcomes in COVID-19 patients. The optimal time window for ECMO initiation remains an area of scientific debate, with arguments for earlier initiation including the expectation of improved outcomes, as patients receive ECMO treatment in a disease state with less irreversible damage. Arguments for later ECMO initiation include prevention of overuse of ECMO and its associated risks in patients who may ultimately recover without ECMO. Data on outcomes related to the timing of ECMO initiation remain inconclusive, and further studies are warranted to this end for COVID-19 patients as well as all patients requiring ECMO [24,25]. Similarly, comorbidities such as cardiovascular disease, diabetes, obesity, and chronic respiratory illness have been shown in previous studies to be outcome predictors in COVID-19, but our study did not find any associations between comorbidities and in-hospital mortality in COVID-19 patients on ECMO [26]. The high predictive power of the model combining the MELD Na, age, and sex did not appear to be generalizable to a cohort of non-COVID-19 patients, suggesting that COVID-19 and non-COVID-19 patients on ECMO have different underlying pathophysiological mechanisms leading to death. This might be related to a greater contribution of multiorgan failure leading to mortality in COVID-19 patients, which is captured by the factors in MELD scoring that relate to renal and hepatic dysfunction. This finding is also in line with previous reports showing that scoring systems predicting mortality in ARDS patients on ECMO may not be generalizable across underlying pathologies [11]. Nonetheless, the MELD scoring system continues to find increasing applications in disease areas beyond its established utility for liver disease, especially cardiac surgery. In the future, refining the MELD score or integrating it with additional biomarkers may further improve its predictive accuracy, enhancing clinical decision-making, optimizing resource allocation, and ultimately improving patient outcomes across a wider spectrum of clinical practice areas.

### Limitations

This study has the limitations inherent to all retrospectively designed studies. The associations between the variables and outcomes cannot be shown to be causal in nature. In addition, small sample sizes in certain subgroups make it difficult to draw meaningful conclusions about these populations. Furthermore, although the MELD scoring system achieved good discriminatory power in our analysis, as it was originally designed for liver disease prognosis, it may not fully account for the complex interplay of factors specific to COVID-19 such as acute respiratory distress syndrome (ARDS) and systemic inflammation. External validation of the scoring model proposed in this study in an independent cohort at a separate institution, ideally compared to other scoring models, is required to assess its broader generalizability.

## 5. Conclusions

A model incorporating the MELD Na, age, and sex has high predictive power for in-hospital mortality in patients with COVID-19 using easily available patient data. The described model draws attention to the contribution of multiorgan failure to mortality and may be clinically useful in critically ill COVID-19 patients both now and in the future, should the virus widely re-emerge.

## Figures and Tables

**Table 1:** MELD score.

Variable	MELD [9]	MELD Na [10]	MELD XI [11]
Bilirubin	x	x	x
INR	x	x	
Creatinine	x	x	x
Sodium		x	

INR = international normalized ratio. MELD = model for end-stage liver disease.

**Table 2:** Characteristics of COVID-19 patients.

Variable	All (*n* = 66)
Sex (% male)	83.3%
Age in years, median (IQR)	58.5 (51–62)
BMI, median (IQR)	29.0 (26–32)
History of CPR (%)	3.0%
History of MI (%)	1.5%
Active smoker (%)	4.5%
Diabetes (%)	18.2%
Hyperlipidemia (%)	4.5%
Hypertension (%)	45.5%
COPD (%)	10.6%
Dialysis at admission (%)	16.7%
Ventilated at admission (%)	72.7%
Symptoms to ECMO in days, median (IQR)	18.0 (11–21)

BMI = body mass index. COPD = chronic obstructive pulmonary disease. CPR = cardiopulmonary resuscitation. ECMO = extracorporeal membrane oxygenation. IQR = interquartile range. MI = myocardial infarction.

**Table 3:** MELD scores by survival status.

Variable	Survivors (*n* = 17)	Non-Survivors (*n* = 49)	*p*-Value
MELD, median (IQR)	9.1 (3.8–12.7)	15.5 (8.6–22.6)	0.006 *
MELD Na, median (IQR)	10.6 (4.8–13.6)	15.7 (11.5–23.6)	0.005 *
MELD XI, median (IQR)	8.2 (5.7–13.4)	13.9 (8.2–22.8)	0.020 *
Creatinine (mg/dL), median (IQR)	0.9 (0.6–1.2)	1.5 (0.9–2.4)	0.008 *
Bilirubin (mg/dL), median (IQR)	0.5 (0.4–1.0)	0.80 (0.5–1.7)	0.124
Sodium (mmol/L), median (IQR)	136 (135–140)	136 (134–139)	0.621
INR, median (IQR)	1.2 (1.2–1.4)	1.3 (1.2–1.5)	0.727

INR = international normalized ratio. IQR = interquartile range. MELD = model for end-stage liver disease. Na = sodium. * statistically significant.

**Table 4:** Variables with discriminatory power for in-hospital mortality.

Variable	AUC	*p*-Value	Optimal Cut-Off	Sensitivity	Specificity
Age (years)	0.680	0.014 *	53.3	58.8%	81.6%
MELD	0.692	0.010 *	10.7	70.6%	67.3%
MELD Na	0.725	0.003 *	13.8	82.4%	59.2%
MELD XI	0.690	0.010 *	13.9	82.4%	51.0%
Creatinine (mg/dL)	0.719	0.004 *	1.4	88.2%	55.1%

MELD = model for end-stage liver disease. Na = sodium. * statistically significant.

## Data Availability

The raw data supporting the conclusions of this article will be made available by the authors upon request due to ethical reasons.
